# *In situ* growth of CuInS_2_ nanocrystals on nanoporous TiO_2_ film for constructing inorganic/organic heterojunction solar cells

**DOI:** 10.1186/1556-276X-8-354

**Published:** 2013-08-16

**Authors:** Zhigang Chen, Minghua Tang, Linlin Song, Guoqiang Tang, Bingjie Zhang, Lisha Zhang, Jianmao Yang, Junqing Hu

**Affiliations:** 1 State Key Laboratory for Modification of Chemical Fibers and Polymer Materials, College of Materials Science and Engineering, Donghua University, Shanghai 201620, China; 2 Analysis and Testing Center, Soochow University, Suzhou 215123, China; 3 College of Environmental Science and Engineering, Donghua University, Shanghai 201620, China; 4 Research Center for Analysis and Measurement, Donghua University, Shanghai 201620, China

**Keywords:** CuInS_2_ film, *In situ* growth, TiO_2_, P3HT, Heterojunction solar cells, 81.15.-z, 84.60.Jt, 73.40.Lq

## Abstract

Inorganic/organic heterojunction solar cells (HSCs) have attracted increasing attention as a cost-effective alternative to conventional solar cells. This work presents an HSC by *in situ* growth of CuInS_2_**(**CIS) layer as the photoabsorption material on nanoporous TiO_2_ film with the use of poly(3-hexylthiophene) (P3HT) as hole-transport material. The *in situ* growth of CIS nanocrystals has been realized by solvothermally treating nanoporous TiO_2_ film in ethanol solution containing InCl_3_ · 4H_2_O, CuSO_4_ · 5H_2_O, and thioacetamide with a constant concentration ratio of 1:1:2. InCl_3_ concentration plays a significant role in controlling the surface morphology of CIS layer. When InCl_3_ concentration is 0.1 M, there is a layer of CIS flower-shaped superstructures on TiO_2_ film, and CIS superstructures are in fact composed of ultrathin nanoplates as ‘petals’ with plenty of nanopores. In addition, the nanopores of TiO_2_ film are filled by CIS nanocrystals, as confirmed using scanning electron microscopy image and by energy dispersive spectroscopy line scan analysis. Subsequently, HSC with a structure of FTO/TiO_2_/CIS/P3HT/PEDOT:PSS/Au has been fabricated, and it yields a power conversion efficiency of 1.4%. Further improvement of the efficiency can be expected by the optimization of the morphology and thickness of CIS layer and the device structure.

## Background

The quest and demand for clean and economical energy sources have increased interest in the development of various solar cells [1], such as Si solar cells [2], Cu(In,Ga)(S,Se)_2_ film solar cells [3-6], organic solar cells [7], and dye-sensitized solar cells (DSSCs) [8-12]. Among these solar cells, DSSCs have been currently attracting widespread scientific and technological interest due to their low cost and high efficiency [8-12]. The typical working principle of DSSCs is based on ultrafast electron injection from a photoexcited dye into the conduction band of TiO_2_ and subsequent dye regeneration and hole transportation to the counter electrode. The power conversion efficiency of DSSCs with organic solvent-based electrolyte has been reported to exceed 11% [9,13,14]. However, DSSCs still suffer from some problems, such as high cost of Ru-based dyes, leakage and/or evaporation from organic solvent-based electrolyte.

For reducing the cost, the use of inorganic semiconductor nanocrystals instead of Ru-based dyes in DSSCs has attracted an enormous interest [15-18]. Semiconductor nanocrystals as the sensitizers have many fascinating advantages, such as high extinction coefficients, large intrinsic dipole moments, and the tuned bandgap [19]. In particular, semiconductor quantum dots have capability of producing multiple electron/hole pairs with a single photon through the impact ionization effect [20]. For depositing semiconductor nanocrystals on TiO_2_ films, two typical approaches have been developed. The first and most common route is the *in situ* synthesis of the nanocrystals on TiO_2_ film, for example, by chemical bath deposition [21] or by successive ionic layer adsorption and reaction (SILAR) [22]. This method provides high surface coverage, but the lack of capping agents leads to a broad size distribution and a higher density of surface defects of nanocrystals, which deteriorates solar cell performance [23]. The second route is the assembly of already-synthesized nanocrystals to TiO_2_ substrates by direct adsorption [24] or linker-assisted adsorption [15]. This *ex situ* approach could achieve better control over the sizes and electronic properties of nanocrystals but suffers from low surface coverage and poor electronic coupling [23]. Up to now, many different semiconductor nanocrystals as the sensitizers have been investigated, including CdSe [17,22,25], CdS [21,26], and PbS [27-29]. Unfortunately, these metal chalcogenide semiconductors are easily oxidized when exposed to light, and this unfavorable situation is even more detrimental when the metal sulfide is in contact with a liquid electrolyte containing sulfur. It is well known that the choice of semiconductors and the method of their deposition play a paramount role in affecting cell efficiency. Therefore, it is still necessary to develop new materials and deposition methods for improving DSSCs with semiconductors as the sensitizers.

On the other hand, for avoiding the sealing problem in DSSCs, many attempts have been made to substitute liquid electrolytes with quasi-solid electrolytes [30] or solid-state hole transporting material (HTM) [31]. Similarly, when semiconductor nanocrystals are used as the sensitizers, some HTMs including poly(3-hexylthiophene) (P3HT) have been developed, resulting in the construction of all-solid-state inorganic/organic heterojunction solar cells (HSCs) [32-36], which possesses the advantages of both DSSCs and traditional organic solar cells. In particular, the efficiency of HSCs with the structure of TiO_2_/Sb_2_S_3_/P3HT has reached 5% [32], which is very close to the efficiencies reported for solid DSSCs using Ru-based molecular dyes. In addition, Sb_2_S_3_ nanocrystals are non-toxic compared with Cd/Pb-based semiconductors. These facts show the great potentiality of all-solid HSCs, which also encourages to further achieve other kind of robust, efficient, and cheap HSCs without toxic component.

Copper indium disulfide (CuInS_2_, abbreviated as CIS) has a small direct bandgap of 1.5 eV that matches well the solar spectrum, a large absorption coefficient (*α* = 5 × 10^5^ cm^−1^), and low toxicity. It has been regarded to be a promising light-absorbing material for film solar cells [4]. As semiconductor sensitizers in DSSCs, CIS nanocrystals have been prepared by different methods and then were coated/adsorbed on TiO_2_ film to construct DSSCs with liquid electrolyte [24,37,38]. In addition, the *in situ* growth of CIS on TiO_2_ film has also been realized, by electrodeposition [16], spin-coating/anneal [39], and SILAR method [40], to construct DSSCs with liquid electrolyte. However, there is little report on solvothermal growth of CIS nanocrystals on TiO_2_ film for the construction of all-solid HSCs. In this paper, we report a facile one-step solvothermal route for the *in situ* growth CIS nanocrystals on nanoporous TiO_2_ film. The effects of reagent concentration on the surface morphology of CIS have been investigated. The all-solid HSC with the structure of FTO/compact-TiO_2_ /nanoporous-TiO_2_/CIS/P3HT/PEDOT:PSS/Au is fabricated, and it exhibits a relatively high conversion efficiency of 1.4%.

## Methods

### Materials

All of the chemicals were commercially available and were used without further purification. Titanium butoxide, petroleum ether, TiCl_4_, CuSO_4_ · 5H_2_O, InCl_3_ · 4H_2_O, thioacetamide, ethanol, methanol, and 1,2-dichlorobenzene were purchased from Sinopharm Chemical Reagent Co., Ltd. (Shanghai, China). TiO_2_ (P25) was obtained from Degussa. Transparent conductive glass (F:SnO_2_, FTO) was purchased from Wuhan Geao Instruments Science & Technology Co., Ltd (Wuhan, Hubei, China). P3HT was bought from Guanghe Electronic Materials Co., Ltd. (Henan, China). The poly(3-4-ethylenedioxythiophene) doped with poly(4-stylenesulfonate) (PEDOT:PSS) solution (solvent, H_2_O; weight percentage, 1.3%) was obtained from Aldrich (St. Louis, MO, USA).

### Preparation of compact and nanoporous TiO_2_ film

A part of FTO glass was chemically etched away in order to prevent direct contact between the two electrodes. A compact (about 100-nm thick) TiO_2_ layer was first deposited onto the FTO glass as follow [41]. FTO glass was dipped into the mixture of titanium butoxide and petroleum ether (2:98 *V*/*V*), taken out carefully, hydrolyzed in air for 30 min, and sintered in oven for 30 min at 450°C.

Then, nanoporous TiO_2_ films were prepared by the doctor-blading technique with TiO_2_ (P25) colloidal dispersion and subsequently sintered at 450°C for 30 min, according to a previous study [42]. This film was soaked into a TiCl_4_ (20 mM in water) solution for 12 h. It was then washed with deionized water and ethanol, dried with air, and sintered again at 450°C for 30 min.

### *In situ* solvothermal growth of CuInS_2_ nanocrystals

CIS layer was *in situ* grown on nanoporous TiO_2_ films by a solvothermal process. In a typical process, thioacetamide (0.24 mmol, 0.02 M) was added into a 12 mL ethanol solution containing InCl_3_ · 4H_2_O (0.01 M) and CuSO_4_ · 5H_2_O (0.01 M) under magnetic stirring, until a clear solution was formed. The resulting solution was transferred into a Teflon-lined stainless steel autoclave with 30-mL capacity. Subsequently, FTO/compact-TiO_2_/nanoporous-TiO_2_ film as the substrate was vertically immersed into the solution. Lastly, the autoclave was kept in a fan-forced oven at 160°C for 12 h. After air-cooling to room temperature, CIS film on non-conductive glass side was scraped off, while CIS film on nanoporous TiO_2_ film side was washed with deionized water and absolute ethanol successively, and dried in air. For comparison, the effects of InCl_3_ · 4H_2_O concentrations (0.01, 0.03, 0.1 M) on the morphologies CIS layer were investigated. The concentration ratio of InCl_3_ · 4H_2_O, CuSO_4_ · 5H_2_O, and thioacetamide was maintained constant (1:1:2) for all the cases.

### Fabrication of all-solid HSC

The P3HT solution (10 mg/mL in 1,2-dichlorobenzene) was spin-coated onto TiO_2_/CIS with 3,000 rpm for 60 s. Then, in order to improve the contact between P3HT and gold, a PEDOT:PSS solution diluted with two volumes of methanol was introduced onto TiO_2_/CIS/P3HT layer by spin-coating at 2,000 rpm for 30 s [32]. In order to form a hybrid heterojunction, the TiO_2_/CIS/P3HT/PEDOT:PSS layer was then annealed at 90°C for 30 min in a vacuum oven. Gold layer as the back contact was prepared by magnetron sputtering with a metal mask, giving an active area of 16 mm^2^ for each device. The resulting HSC has a structure of FTO/compact-TiO_2_/nanoporous-TiO_2_/CIS/P3HT/PEDOT:PSS/Au.

### Characterization and photoelectrical measurements

The sizes and morphologies of the sample were investigated by field emission scanning electron microscopy (FE-SEM; S-4800, Hitachi, Chiyoda-ku, Japan). During SEM measurement, energy dispersive spectroscopy (EDS; Quantax 400, Bruker AXS, Inc., Madison, WI, USA) line scan was also performed to locate and determine the distribution of different layer in the composite film. The X-ray diffraction (XRD; D/max-g B, Rigaku, Shibuya-ku, Japan) measurement was carried out using a Cu Kα radiation source (*λ* = 1.5418 Å). An ultraviolet/visible (UV-vis) spectrophotometer (U-3010 spectrophotometer, Hitachi, Chiyoda-ku, Japan) was used to carry out the optical measurements. The photocurrent density/voltage curves of HSC was measured under illumination (100 mW cm^−2^) using a computerized Keithley Model 2400 Source Meter unit (Keithley Instruments Inc., Cleveland, OH, USA) and a 300-W xenon lamp (Newport 69911, Newport-Oriel Instruments, Stratford, CT, USA) serving as the light source.

## Results and discussion

**Figure 1:**
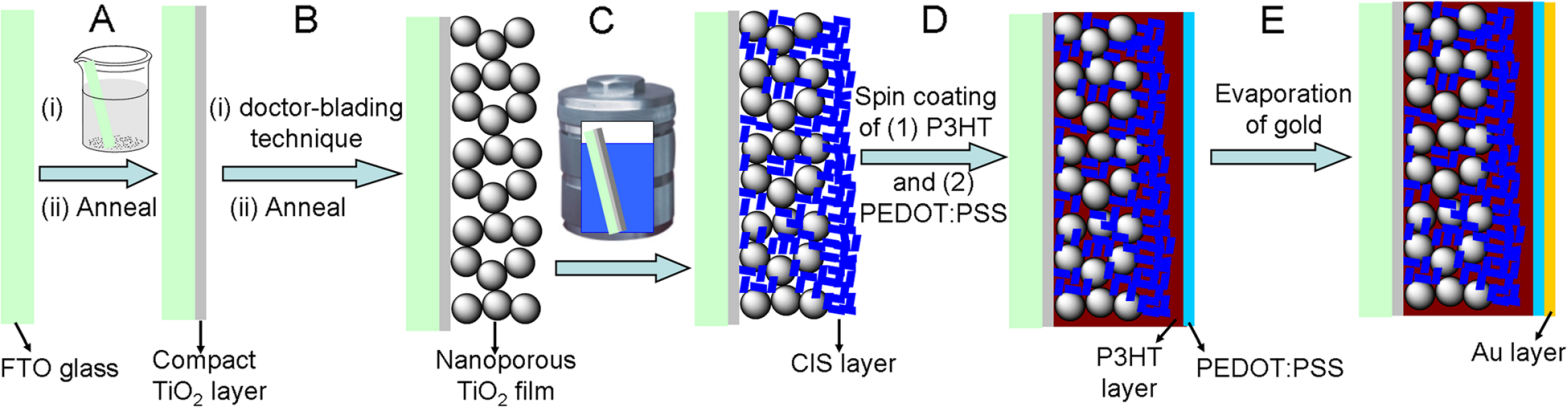
**Schematic illustration of the fabrication process of FSCs. (A)** preparation of compact TiO_2_ film; **(B)** preparation of nanoporous TiO_2_ film; **(C)** solvothermal growth of CIS layer; **(D)** spin-coating of P3HT and PEDOT:PSS; **(E)** evaporation of gold layer.

**Figure 2:**
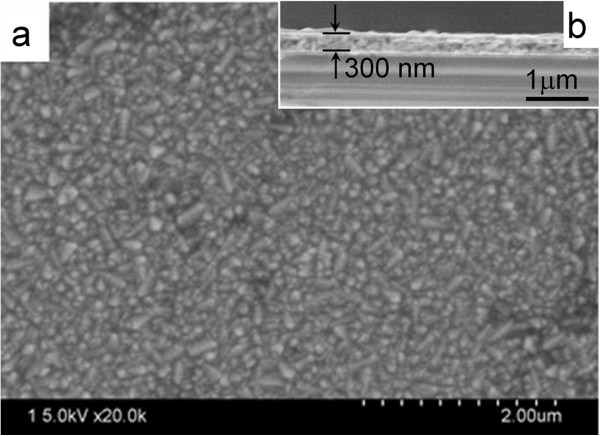
**Surface (a) and cross-sectional (b) SEM images of dense TiO**_**2 **_**layer.**

Herein, the fabrication of all-solid HSC with the structure of FTO/compact-TiO_2_ /nanoporous-TiO_2_/CIS/P3HT/PEDOT:PSS/Au involved five steps, as demonstrated in Figure 1. The first step was to prepare a compact TiO_2_ layer by a dip-coating-anneal process (Figures 1 (step A) and 2), according our previous study [41]. SEM images (Figure 2) confirm the formation of a dense TiO_2_ layer on FTO glass, and this TiO_2_ layer has a thickness of about 300 nm. The presence of compact TiO_2_ layer can not only improve the ohmic contact but also avoid short circuiting and/or loss of current by forming a blocking layer between FTO and P3HT in the HSC.

**Figure 3:**
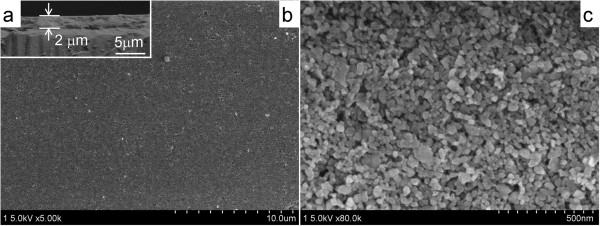
**SEM images of nanoporous TiO**_**2 **_**film: (a) cross-sectional, (b) low-, and (c) high-magnification SEM images of the surface.**

The second step was to fabricate nanoporous TiO_2_ film on FTO/compact-TiO_2_ by a classic doctor-blading-anneal technique with TiO_2_ (P25) colloidal dispersion (Figures 1 (step B) and 3) [42]. Such nanoporous TiO_2_ film has a thickness of about 2 μm, as revealed by cross-sectional SEM image (Figure 3a). In addition, one can find that the surface of nanoporous TiO_2_ film is uniform and smooth without crack (Figure 3b). High-resolution SEM (Figure 3c) reveals the TiO_2_ film to be composed of a three-dimensional network of interconnected particles with an average size of approximately 30 nm. It also can be found that there are many nanopores in the TiO_2_ film, which facilitates to absorb dye and/or other semiconductor nanocrystals.

**Figure 4:**
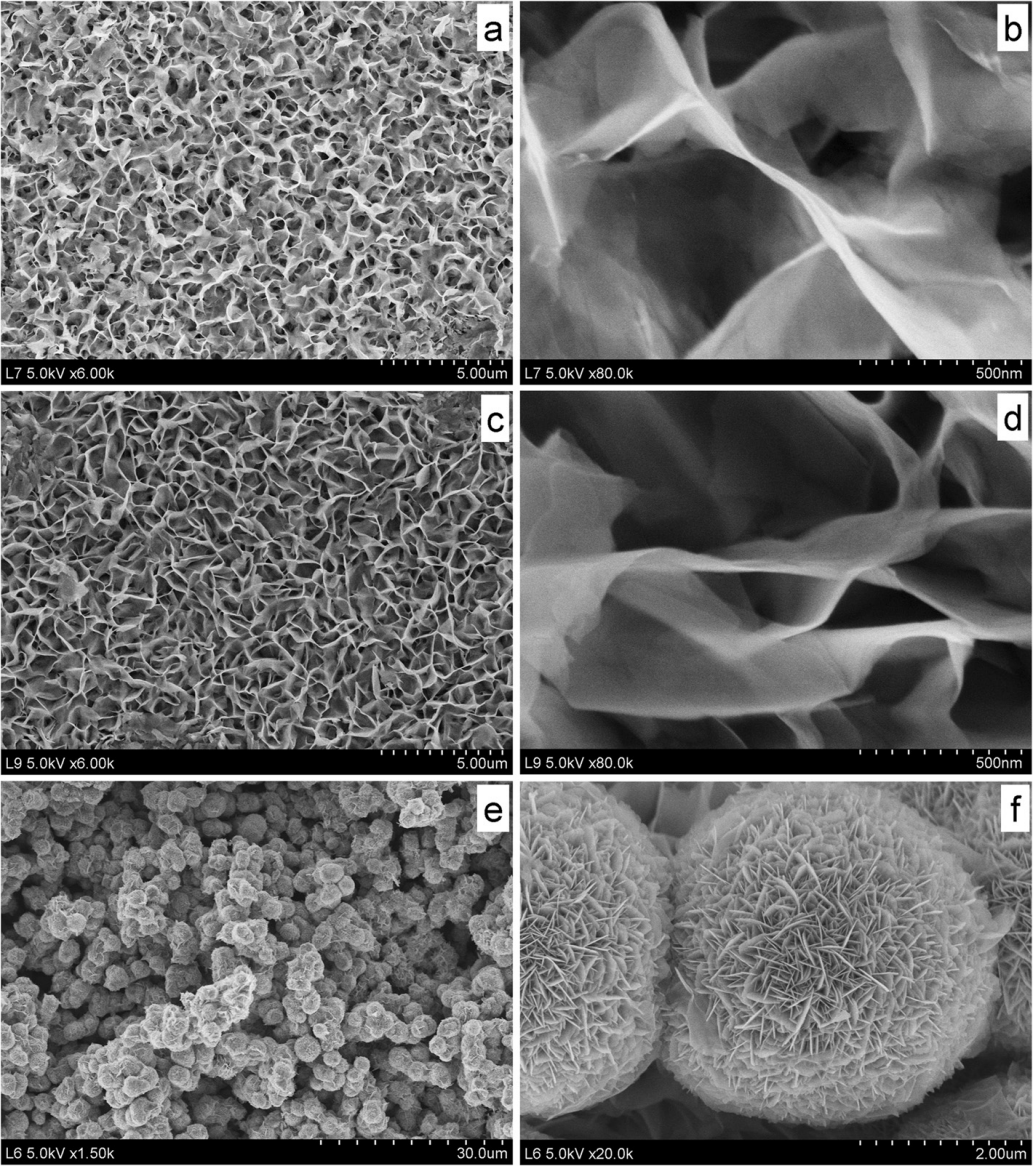
**SEM images of CIS layer on TiO**_**2 **_**film, obtained by a solvothermal treatment.** At 160°C for 12 h with different InCl_3_ concentration: **(a**,**b)** 0.01 M; **(c**,**d)** 0.03 M; **(e**,**f)** 0.1 M.

The third step was to *in situ* grow CIS nanocrystals on nanoporous TiO_2_ film by the classic solvothermal process (Figure 1C), where FTO/compact-TiO_2_/nanoporous-TiO_2_ film as the substrate was vertically immersed into the ethanol solution containing InCl_3_, CuSO_4_, and thioacetamide with constant concentration ratio (1:1:2) as the reactant, and the solution was solvothermally treated at 160°C for 12 h. It has been found that reactant concentrations play a significant role in the controlled growth of CIS films in our previous study [4]. Thus, the effects of reactant concentration (such as InCl_3_ concentration: 0.01, 0.03, 0.1 M) on the surface morphologies of CIS layer were investigated by SEM observation. Figure 4 gives the typical morphologies of CIS films prepared with different InCl_3_ concentration. When InCl_3_ concentration is low (0.01 or 0.03 M), a large amount of high-ordered potato chip-shaped CIS nanosheet arrays are densely packed and uniformly covered over the entire surface of FTO/compact-TiO_2_/nanoporous-TiO_2_ film (Figure 4a,c), which is similar to the *in situ* growth of CIS on Cu foil [4]. More detailed nanostructure about CIS film can be observed using high-magnification SEM images (Figure 4b,d), where individual CIS nanosheet displays a crooked shape with a thickness of approximately 10 nm and length of approximately 2 μm. These CIS potato chip-shaped nanosheets are assembled and intermeshed with each other, forming a continuous net-like flat film. It should be noted that CIS chips may be too big to separate efficiently electron/hole pairs in the application of HSCs. As InCl_3_ concentration increased to 0.1 M, CIS flower-shaped superstructures with an average diameter of 3 μm spread over the whole FTO/compact-TiO_2_/nanoporous-TiO_2_ film (Figure 4e). In fact, as shown in the SEM image with higher magnification (Figure 4f), CIS superstructures are composed of ultrathin nanoplates as ‘petals’ with an average thickness of approximately 10 nm and length of approximately 0.6 μm. These ‘petals’ were aligned perpendicularly to the spherical surface with clearly oriented layers, pointing toward a common center. In addition, many hierarchical nanopores could be found among spherical superstructures and also among their ‘petals,’ which would improve the physicochemical properties.

**Figure 5:**
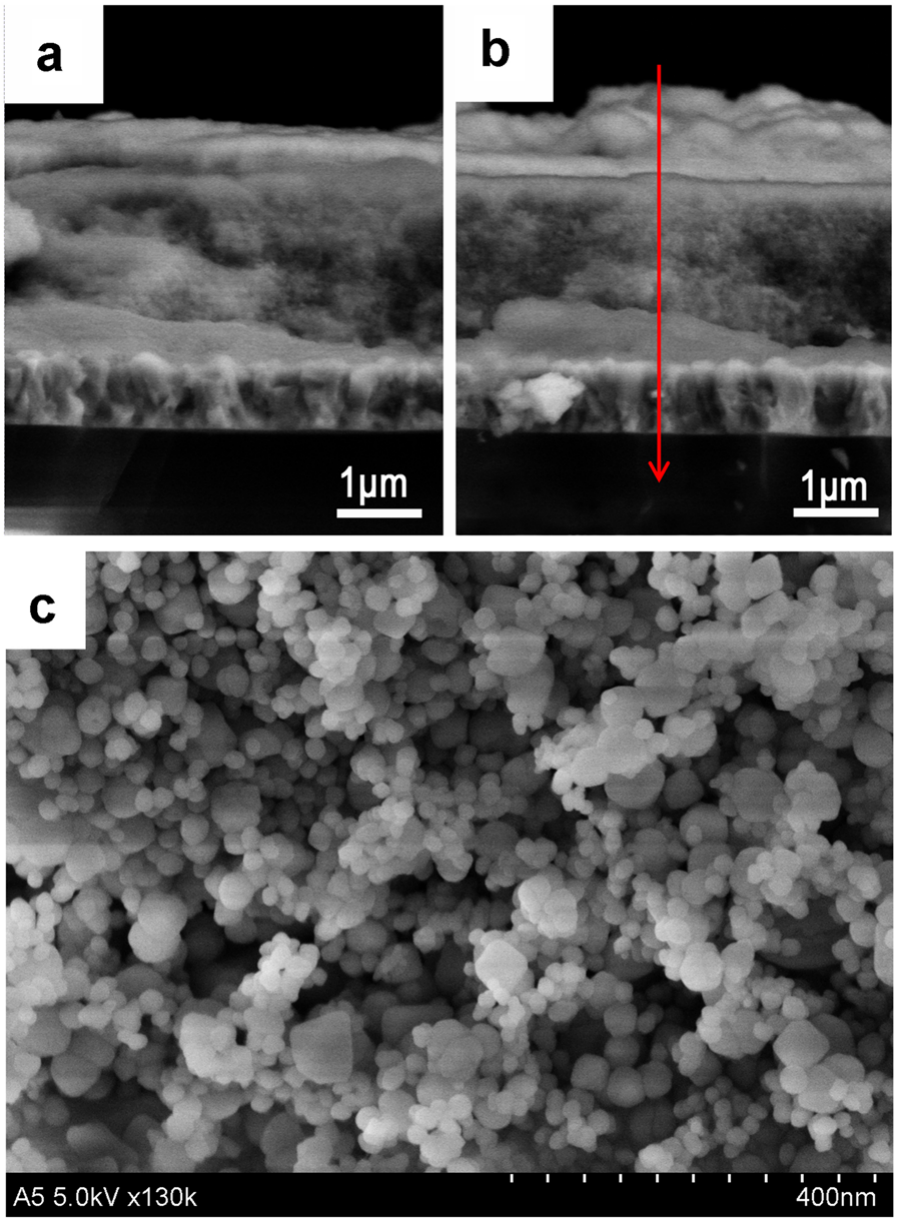
**Cross-sectional SEM images of samples with CIS film prepared from (a) 0.03 M or (b,c) 0.1 M InCl**_**3**_**.**

**Figure 6:**
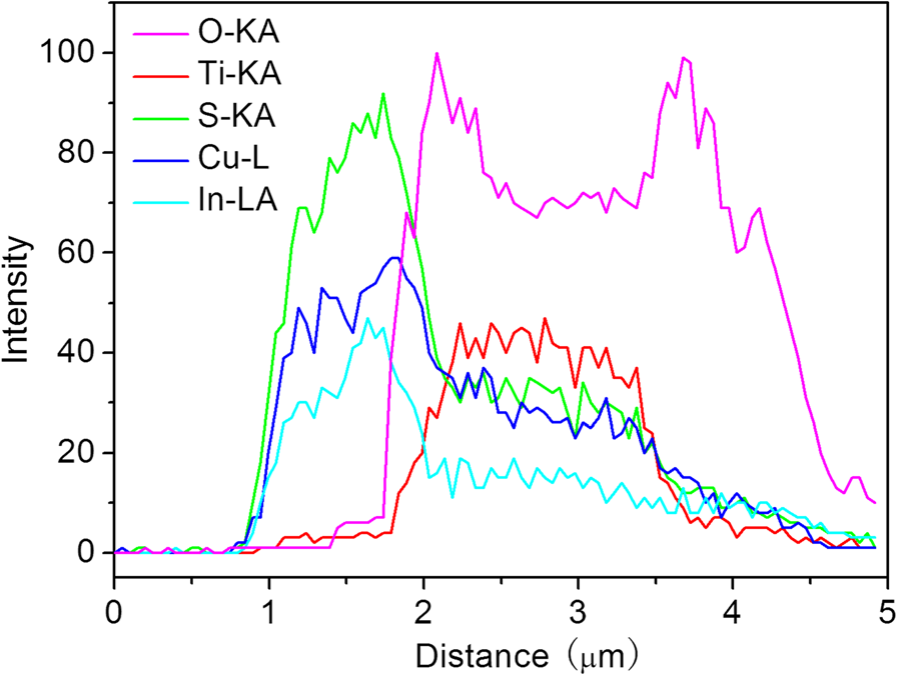
**EDS line scan analysis along the red line indicated in the SEM image (Figure**5**b).**

Subsequently, TiO_2_/CIS film samples were further characterized. To confirm the structure and composition of samples with CIS prepared with 0.03 or 0.1 M InCl_3_, their cross-sectional morphologies were investigated. Obviously, a new layer can be found on the nanoporous TiO_2_ film, and the pores in TiO_2_ film have been partly filled with some nanoparticles (Figure 5a,b). Since CIS flower-shaped superstructure prepared from 0.1 M InCl_3_ is composed of ultrathin nanoplates as ‘petals’ and should be more suitable for HSC, we further analyzed the microstructure and local atomic composition of this film sample. Figure 5c shows the high-magnification SEM image in the middle of the cross section. Obviously, there are two kinds of nanoparticles. One has the relatively large diameter of about 20 to 50 nm, and it should be TiO_2_ nanoparticles, according to the SEM image (Figure 3c) of TiO_2_ film substrate. The other has the relatively small diameter of about 10 nm, and it should be CIS nanoparticles which were filled into the pores of TiO_2_ films. Furthermore, the red arrow on the SEM image shown in Figure 5b indicates the scanning path of an electron beam, and a clear presentation of the elemental distribution is given by a plot of the EDS line scan signal versus the distance along the film (Figure 6). Overall, EDS line scan profile shows that the signal peaks of the Cu, In, and S elements locate at the first region, indicating the presence of CIS. Subsequently, all signals from Cu, In, and S elements exhibit an obvious drop and then a fairly flat upon further increase of scanning distance in the middle region. On the contrary, in this middle region, the signal from Ti and O elements increase rapidly and then exhibit a fairly flat upon further increase of scanning distance. The clear distinct difference in the spatial profiles from CIS and TiO_2_ is well consistent with well-defined structures and SEM images, confirming that there is a CIS layer on the top of TiO_2_ film, and the pores of TiO_2_ film have been filled by CIS nanoparticles.

**Figure 7:**
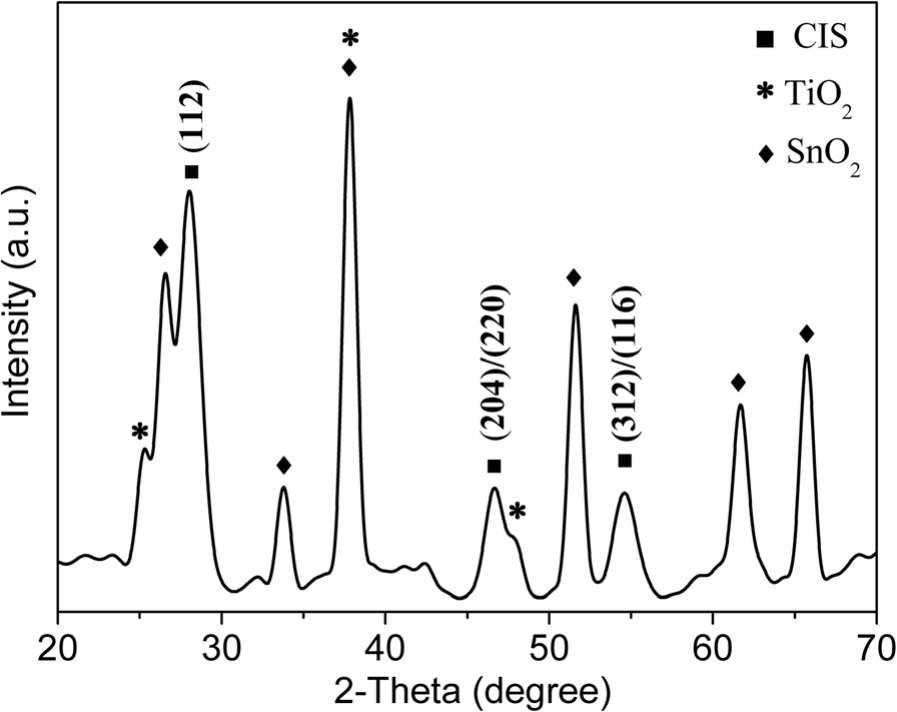
**XRD pattern of TiO**_**2**_**/CIS film sample, where CIS film was prepared from 0.1 M InCl**_**3**_**.**

**Figure 8:**
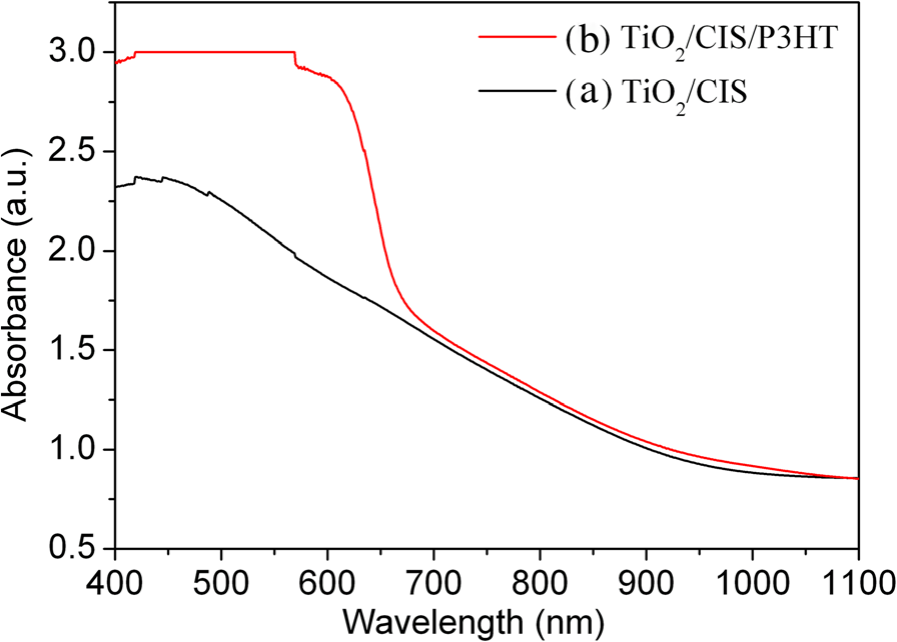
**UV-vis/NIR absorption spectra.** TiO_2_/CIS **(a)** and TiO_2_/CIS/P3HT **(b)** film samples.

Furthermore, the phase and optical property of TiO_2_/CIS film sample with CIS prepared with 0.1 M InCl_3_ were investigated. Figure 7 shows the typical XRD pattern. Besides those existing peaks from SnO_2_ (2*θ*: 26.6°, 33.8°, 37.8°, 51.7°, 61.8°, 65.8°; from FTO substrate) and TiO_2_ film (2*θ*: 25.3°, 37.8°, 48.0°), the diffraction peaks at 27.8°, 46.5°, and 55.1° are assigned to (112), (204)/(220), and (312)/(116) planes of CIS, respectively, which are consistent with our previous study [4] and the data obtained from JCPDS card no. 85-1575. This fact confirms that CIS layer is well crystallized and has chalcopyrite structure. Furthermore, the optical absorption of TiO_2_/CIS film was measured using a UV-vis spectrometer, as shown in Figure 8 (line A). This spectrum presents strong adsorption within a broad range between 400 and 800 nm, which is the characteristic absorption of CIS and consistent with our previous study [4].

The fourth step was to in turn deposit P3HT and PEDOT:PSS layer on FTO/compact-TiO_2_/nanoporous-TiO_2_/CIS film by the spin-coating process (Figure 1 (step D)). After the coating of P3HT, the photoabsorption of the film increases obviously in the range of 400 to 700 nm, as shown in Figure 8 (line B), since P3HT solution exhibits a wide and strong absorption with peak at about 445 nm [43]. This fact also indicates the efficient deposition of P3HT in/on TiO_2_/CIS film. It should be noted that there are plenty of macro-pores among superstructures, nanopores inside CIS flower-shaped superstructures, and nanopores in TiO_2_ film due to the insufficient filling. The hierarchical combination of smaller nanopores and larger macro-pores can be considered as transport paths [41]. It can be expected that P3HT solution can easily enter the deep layer of FTO/compact-TiO_2_/nanoporous-TiO_2_/CIS film through the transport paths, when they are coated onto its surface during the spin-coating process. This should lead to better effects of wetting and pore-filling and thus better interfacial contact among P3HT, CIS, and TiO_2_, probably resulting in more efficient separation of photoinduced electron/hole pairs and thus higher photocurrent.

**Figure 9:**
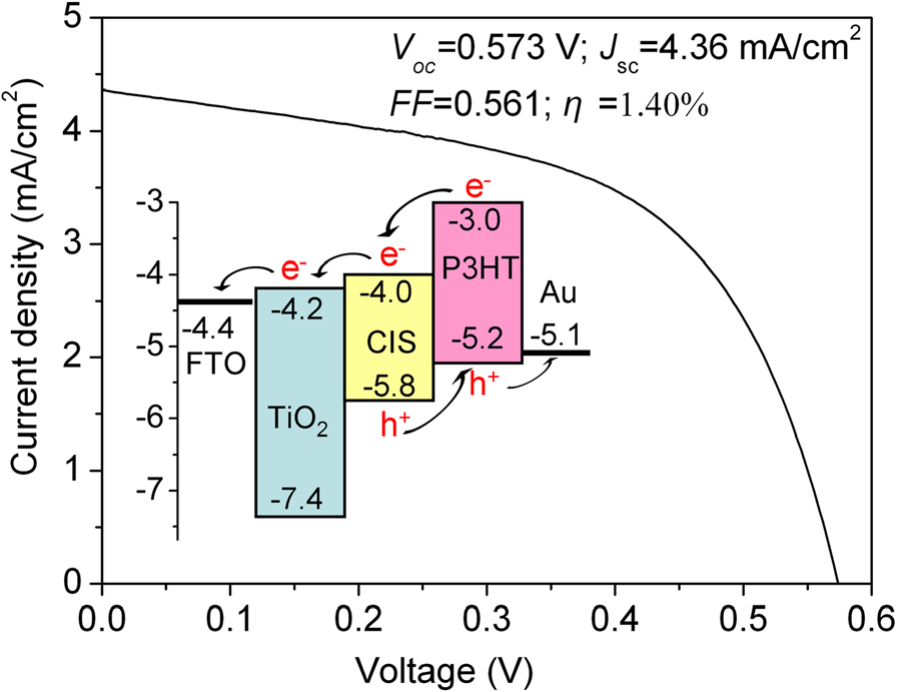
**J-V characteristic curve of the HSC.** The inset is band alignment among TiO_2_, CIS, and P3HT.

The last step was to prepare gold electrode with the thickness of 100 nm on the resulting film for completing the construction of HSC (Figure 1 (step E)). Photocurrent density/voltage characteristics of the resulted HSC are shown in Figure 9. The cell exhibits an open circuit voltage (*V*_oc_) of 0.573 V, a short-circuit current density (*J*_sc_) of 4.36 mA/cm^2^, and a fill factor (*FF*) of 0.561, yielding an overall energy conversion efficiency (*η*) of 1.40%. This conversion efficiency has been greatly improved, compared with that (typically 0.1% to 1.0%) of TiO_2_/P3HT hybrid HSCs in the absence of dye or PCBM [44-47]. There are chiefly three reasons for the improvement. The first reason is the good band alignment among TiO_2_, CIS, and P3HT (the inset of Figure 9), resulting in the fact that exciton dissociation and charge transfer at the interface are energetically favorable. The second reason should be attributed to the strong photoabsorption of CIS and P3HT, as revealed in Figure 8, since the successful sensitization of TiO_2_ by CIS layer has been well demonstrated by the previous studies [24,38,40]. The last reason results from the good interfacial contact among P3HT, CIS, and TiO_2_ due to hierarchical pores in CIS and TiO_2_ layer, as demonstrated in Figures 4 and 5. In addition, it should be noted that our cell efficiency (1.4%) is relatively low compared with that (3% to 5%) of HSC with the structure of TiO_2_/Sb_2_S_3_/P3HT [32,36,48,49], which probably results from the large size of CIS, unoptimized cell structure, etc. Therefore, further improvement of the efficiency could be expected by the optimization of the morphology and thickness of CIS layer and the device structure.

## Conclusions

In summary, an *in situ* growth of CIS nanocrystals has been demonstrated by solvothermally treating nanoporous TiO_2_ film in ethanol solution containing InCl_3_ · 4H_2_O, CuSO_4_ · 5H_2_O, and thioacetamide with a constant concentration ratio of 1:1:2. When InCl_3_ concentration is 0.1 M, there is a CIS layer on the top of TiO_2_ film, and the pores of TiO_2_ film have been filled by CIS nanoparticles. An HSC with the structure of FTO/TiO_2_/CIS/P3HT/PEDOT:PSS/Au has been fabricated, and it yields a power conversion efficiency of 1.4%. Further improvement can be expected by optimizing CIS layer and the cell structure.

## Competing interests

The authors declare that they have no competing interests.

## Authors’ contributions

ZC designed the experiment and wrote the article. ZC, MT, and LS carried out the laboratory experiments. GT, BZ, LZ, JY, and JH assisted the technical support for measurements (SEM, EDS, XRD, UV–vis/NIR absorption, and I-V) as well as the data analysis. All authors read and approved the final manuscript.
